# Synthesis, molecular docking and biological evaluation of bis-pyrimidine Schiff base derivatives

**DOI:** 10.1186/s13065-017-0322-0

**Published:** 2017-09-18

**Authors:** Sanjiv Kumar, Siong Meng Lim, Kalavathy Ramasamy, Mani Vasudevan, Syed Adnan Ali Shah, Manikandan Selvaraj, Balasubramanian Narasimhan

**Affiliations:** 1 Faculty of Pharmaceutical Sciences, Maharshi Dayanand University, Rohtak, 124001 India; 2 Faculty of Pharmacy, Universiti Teknologi MARA (UiTM), Puncak Alam Campus, 42300 Bandar Puncak Alam, Selangor Darul Ehsan Malaysia; 3 Collaborative Drug Discovery Research (CDDR) Group, Pharmaceutical Life Sciences Community of Research, Universiti Teknologi MARA (UiTM), 40450 Shah Alam, Selangor Darul Ehsan Malaysia; 4 Department of Pharmacology and Toxicology, College of Pharmacy, Qassim University, Buraidah, 51452 Kingdom of Saudi Arabia; 5 Atta-ur-Rahman Institute for Natural Products Discovery (AuRIns), Universiti Teknologi MARA, Puncak Alam Campus, 42300 Bandar Puncak Alam, Selangor Darul Ehsan Malaysia; 6 Integrative Pharmacogenomics Institute (iPROMISE), Universiti Teknologi MARA (UiTM), Puncak Alam Campus, 42300 Bandar Puncak Alam, Selangor Darul Ehsan Malaysia

**Keywords:** Bis-pyrimidine Schiff bases, Antimicrobial, Anticancer, Molecular docking

## Abstract

**Background:**

Heterocyclic pyrimidine nucleus, which is an essential base component of the genetic material of deoxyribonucleic acid, demonstrated various biological activities. A series of bis-pyrimidine Schiff bases were synthesized and screened for its antimicrobial and anticancer potentials. The molecular docking study was carried to find the interaction between active molecules with receptor.

**Results:**

The structures of synthesized bis-pyrimidine Schiff bases were confirmed by spectral studies. The synthesized bis-pyrimidine derivatives were evaluated for their antimicrobial activity (MIC = µmol/mL) against selected Gram positive; Gram negative bacterial and fungal strains by tube dilution method. The anticancer activity (IC_50_ = µmol/mL) of the synthesized compounds was determined against human colorectal carcinoma (HCT116) cancer cell line by Sulforhodamine B (SRB) assay. Molecular docking studies provided information regarding the binding mode of active bis-pyrimidine Schiff bases with the cyclin-dependent kinase 8 (CDK8) receptor.

**Conclusions:**

The antimicrobial screening results indicated that compounds, **q1** (MIC_bs_ = 0.83 µmol/mL), **q16** (MIC_an_ = 1.54 µmol/mL and MIC_ec_ = 0.77 µmol/mL), **q1** and **q19** (MIC_ca_ = 0.41 µmol/mL) and **q20** (MIC = 0.36 µmol/mL) are the most active ones. Compounds **q1** (IC_50_ = 0.18 µmol/mL) have emerged as potent anticancer molecule against human colorectal carcinoma cancer cell line than the reference drug, 5-fluorouracil. Molecular docking studies indicated that compound **q1** (the most active molecule) has the maximum hydrogen bond interaction (four) and π–π stacking (three) network among the bis-pyrimidine Schiff bases.

**Graphical abstract:**
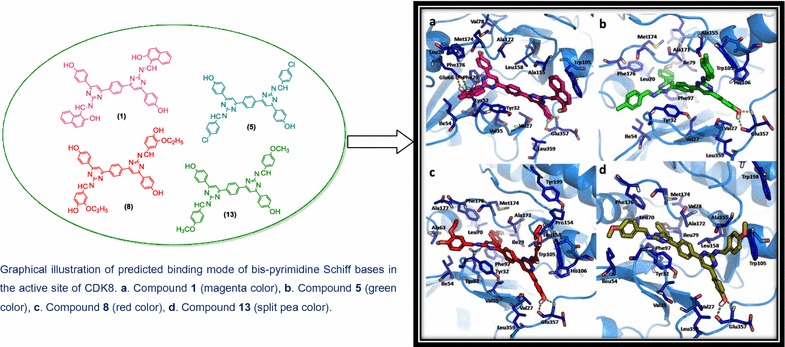
Graphical illustration of predicted binding mode of bis-pyrimidine Schiff bases in the active site of CDK8.** a**. Compound** 1** (magenta color),** b**. Compound** 5** (green color),** c**. Compound** 8** (red color),** d**. Compound** 13** (split pea color).

## Background

Development of novel antimicrobial molecules may provide additional options for the treatment of various microbial infections which affects millions of people worldwide. Cancer is one of the most serious health problems all over the world and one of the leading causes of death, so there is an urgent ongoing need for discovery a highly effective new molecule for cancer treatment with fewer side effects. Heterocyclic pyrimidine nucleus, which is an essential base component of the genetic material of deoxyribonucleic acid, demonstrated various biological activities viz. antimicrobial [1], anticancer [2], antiviral [3], anti-inflammatory [4], antifungal [5], analgesic [6], anticonvulsant [7], antioxidant [8], antitubercular, antimalarial [9] and antileishmanial [10] etc.

**Fig. 1:**
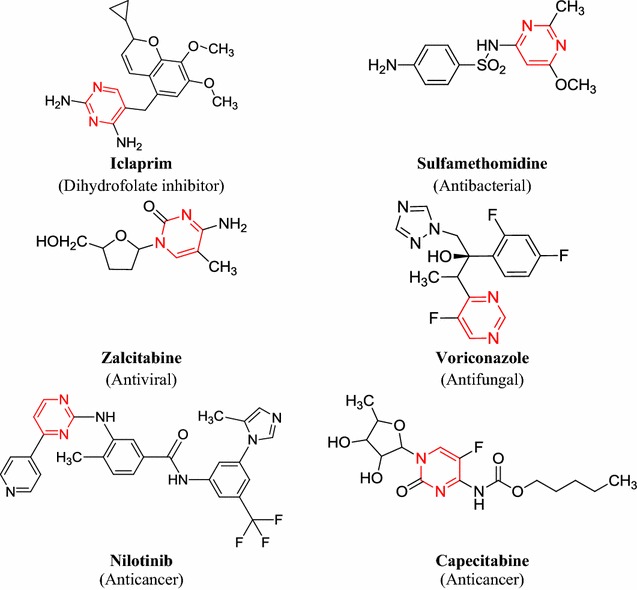
Marketed preparations of pyrimidine molecules

Molecular docking technique is routinely used in modern drug discovery for understanding the drug-receptor interaction. This technique has frequently been used to predict the binding affinity and orientation of small drug molecules at the target site. The two aims of docking studies are accurate structural modelling and correct prediction of activity. Macromolecular docking studies provides the most detailed possible view of drug–receptor interaction and has created a new rational approach to drug design, where the structure of drug is designed based on its fit to 3D structures of a receptor site [11]. Some marketed drugs contains pyrimidine moiety presented in Fig. 1.

**Fig. 2:**
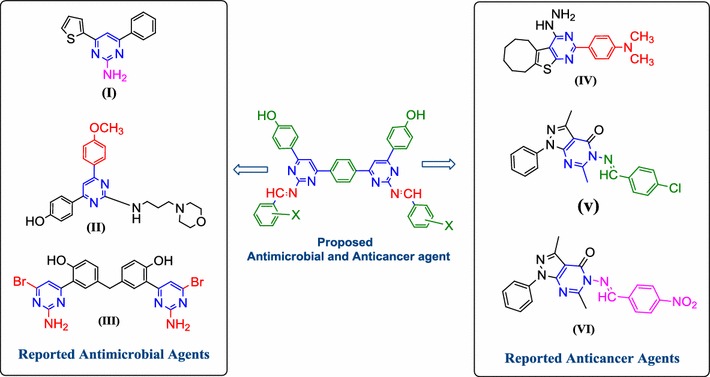
Design of heterocyclic bis-pyrimidine derivatives for antimicrobial and anticancer activity based on literature

Literature reports reveal that –NH_2_ group at the 2nd position of pyrimidine enhanced the antimicrobial potential [**I**] of pyrimidines [12]. *p*-Methoxyphenyl nucleus at 6th position on pyrimidine nucleus [13] showed antimicrobial activity [**II**]. The Ar–Br group on 4th position of pyrimidine nucleus [**III**] improved the antimicrobial potential [14], *p*-dimethyl amino phenyl nucleus [**IV**] attached on the pyrimidine nucleus improved the anticancer potential against HCT-116 cell line [15], *p*-chloro and *p*-nitrobenzylideneamino at the 5th position of pyrimidine ring [**V**–**VI**] improved the anticancer potential of pyrimidine [16]. The aforementioned findings are summarized in Fig. 2.

Prompted by aforementioned facts, in the present work we have planned to synthesize bis-pyrimidine Schiff bases of 4,4′-(6,6′-(1,4-phenylene)bis(2-aminopyrimidine-6,4-diyl))diphenol and evaluate their antimicrobial and anticancer potentials along with molecular docking studies.

## Results and discussion

### Chemistry

**Scheme 1:**
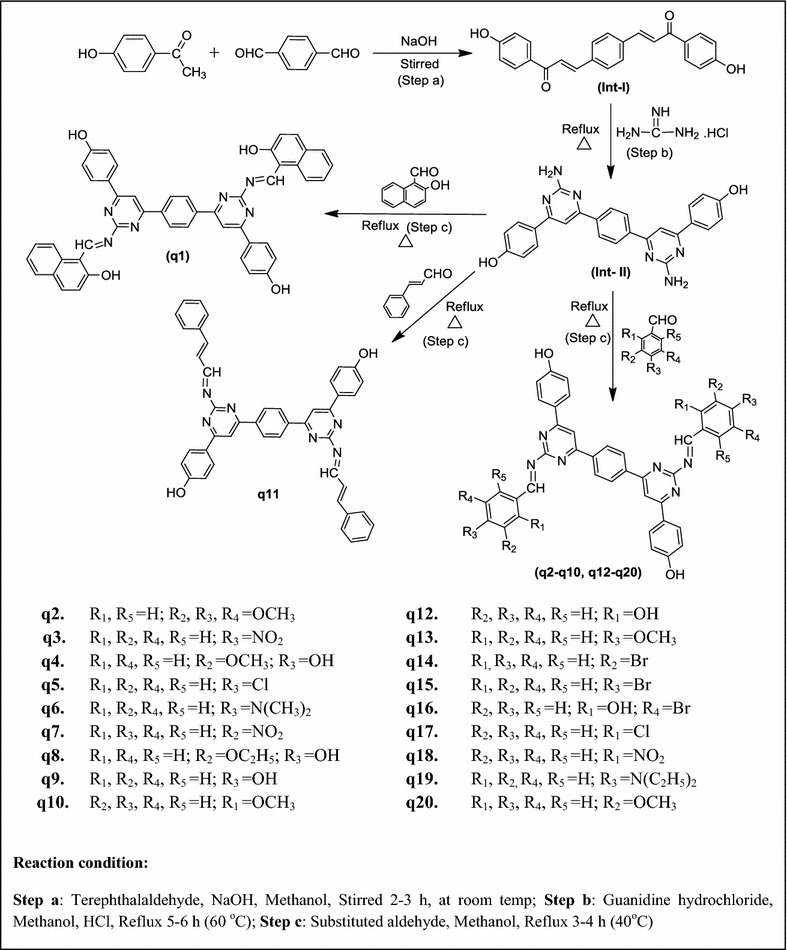
Synthetic route followed for the synthesis of bis-pyrimidine Schiff bases

The synthetic work is based on Claisen-Schmidt condensation (Scheme 1). Initially, the bis-chalcone was synthesized by the reaction of 1-(4-hydroxyphenyl)ethanone and terephthalaldehyde. The cyclization of bis-chalcone (intermediate-**I**) to yield bis-pyrimidine (intermediate-**II**) was effected with guanidine hydrochloride. The reaction of bis-pyrimidine (intermediate-**II**) with corresponding substituted aldehyde resulted in the formation of title compounds (**q1**–**q20**). The poor % yield of some of the synthesized compounds may be attributed to any one or more of the following reasons: (1) The reaction may be reversible and position of equilibrium is unfavorable to the product; (2) The incursion of side reactions leading to the formation of by-products; (3) The premature work-up of the reaction before its completion; (4) The volatilization of products during reaction or work-up; (5) The loss of product due to incomplete extraction, inefficient crystallization or other work-up procedures; (6) The presence of contaminants in the reactants or reagents leading to a less efficient reaction [17]. The synthesized compounds were characterized by the determination of their physicochemical and spectral characteristics. The chemical structures of the synthesized bis-pyrimidine Schiff bases (**q1**–**q20**) were established by ^1^H/^13^C-NMR, FT-IR, mass spectral studies and elemental analysis. The IR spectrum of bis-chalcone (**I**) showed the characteristic band at 1693 cm^−1^ which indicated the presence of a –C=O group and characteristic bands at 3088 and 1427 cm^−1^ for the presence of C–H and C=C group in aromatic ring, respectively. The existence of Ar–OH group in bis-chalcone (**I**) was displayed by the existence Ar–OH stretches in the scale of 3363 cm^−1^ and characteristic bands at 2864 and 1497 cm^−1^ indicated the presence of C–H and C=C group in alkyl chain, respectively. Bis-pyrimidine (**II**) showed the characteristic IR bands at 3058 and 1537 cm^−1^ for the presence of C–H and C=C group in aromatic ring, respectively and characteristic bands at 3331 and 1604 cm^−1^ for the presence of –NH_2_ and N=CH str. The structure of the bis-chalcone and its cyclized products were further confirmed by the corresponding ^1^H-NMR spectra. The ^1^H-NMR spectrum of bis-chalcone **I** showed two doublets at 7.59 ppm (*J* = 15.1 Hz) and 8.06 ppm (*J* = 15.1 Hz) indicating that the CH=CH group in the enone linkage is in a *trans*-conformation. The ^1^H-NMR spectrum of intermediate-**II** showed a multiplet signals between 7.65 and 8.26 δ ppm confirming the cyclisation of the bis-chalcone to give bis-pyrimidine ring. The ^1^H-NMR spectrum of compound intermediate-**II** showed a sharp singlet at 7.26 δ ppm due to the NH_2_ protons and it also showed a sharp singlet at 7.60 δ ppm due to HC=C group, which confirmed the cyclization of the bis-chalcone into a bis-pyrimidine ring. The impression of IR absorption band at 3387 − 2237 cm^−1^ in the spectral data of synthesized derivatives (**q1**–**q20**) displayed the presence of Ar–OH category on the aromatic nucleus substituted at the *ortho, meta* and *para*-position of the synthesized derivatives. The IR absorption band in the scale of 690–515 cm^−1^ corresponds to the C–Br stretching of aromatic-bromo derivatives (**q14**, **q15** and **q16**). The existence of Ar–NO_2_ category in derivatives **q3**, **q7** and **q18** was displayed by the existence of symmetric and asymmetric Ar–NO_2_ stretches in the scale of 1365 − 1335 and 1550 − 1510 cm^−1^ respectively. The existence of an arylalkyl ether category (Ar–OCH_3_) in derivatives, **q2**, **q4**, **q10**, **q13** and **q20** are established by the existence of an IR absorption band around 3150 − 3050 cm^−1^. Further, the existence of halogen group in compounds **q5** and **q17** is indicated by the existence of Ar–Cl stretching vibrations at 600–800 cm^−1^. The impression of IR stretching vibration at 3100–3000 and 1580–1600 cm^−1^ in the spectral data of synthesized derivatives (**q1**–**q20**) specified the existence of C–H and C=C group, respectively. The appearance of IR stretching 1604–1700 cm^−1^ in the spectral data of synthesized derivatives (**q1**–**q20**) specified the existence of N=CH group. The impression of IR stretching at 1630 cm^−1^ in the spectra of intermediate specified the existence of C=O group. The multiplet signals between 6.75 and 8.22 δ ppm in ^1^H-NMR spectra is indicative of aromatic proton of synthesized derivatives. The compounds, **q2**, **q4**, **q10**, **q13** and **q20** showed singlet at 3.71–3.82 δ ppm due to the existence of OCH_3_ of Ar–OCH_3_. All compounds showed singlet at 7.51–8.43 δ ppm due to the existence of N=CH in pyrimidine ring. Compounds showed singlet at 7.70–7.74 δ ppm due to the existence of –CH in pyrimidine ring. Compound **q6** showed singlet at 2.89 δ ppm due to existence of –N(CH_3_)_2_ at the *para* position. The compound **q19** showed quadrate at 3.41 δ ppm and triplet at 1.13 δ ppm due to presence of –N(C_2_H_5_)_2_ at *para* position. The elemental analysis studies of the synthesized bis-pyrimidine Schiff bases were found within ±0.4% of the theoretical results. Finally, the ^13^C-NMR spectra of the bis-chalcone and the cyclized bis-pyrimidine were recorded in DMSO-*d*_6_ and the spectral signals were in good agreement with the proposed molecular structure of the synthesized compounds. ^13^C-NMR spectral interpretation details synthesized compounds are given in the experimental section.

### In vitro antimicrobial activity

Antimicrobial screening of synthesized derivatives against Gram +ve bacterial species: *Staphylococcus aureus*, *Bacillus subtilis*, the Gram −ve bacterium *Escherichia coli* and fungal species: *Aspergillus niger* and *Candida albicans* was done by tube dilution technique. Antimicrobial activity results indicated (Table 1) particularly; compounds **q1**, **q16**, **q19** and **q20** have shown more promising antimicrobial activity as compared to standard drugs norfloxacin (antibacterial) and fluconazole (antifungal) while other derivatives are moderately active. In the case of Gram +ve antibacterial study, compound **q1** was found to be most potent one against *B. subtilis* with MIC value of 0.83 µmol/mL and compound **q20** showed significant activity against *S. aureus* with MIC value of 0.36 µmol/mL. In the case of Gram −ve bacterial study, compound **q16** displayed appreciable antibacterial activity against *E. coli.* The antifungal activity results indicated that compounds **q1** and **q19** (MIC_*ca*_ = 0.41 µmol/mL) and compound **q16** (MIC_*an*_ = 1.54 µmol/mL) were found to be most effective ones against *C. albicans* and *A. niger*, respectively. The most active synthesized bis-pyrimidine Schiff base derivatives **q19** and **q20** may be taken as lead compounds to discover novel antimicrobial agent.

**Table 1:** Antimicrobial and anticancer activities of synthesized bis-pyrimidine Schiff bases

Compounds	Minimum inhibitory concentration (MIC = µmol/mL)					IC_50_ = µmol/mL
	Bacterial species			Fungal species		(HCT116) cancer cell line
	Gram positive		Gram negative	*C. albicans* (MTCC 227)	*A. niger* (MTCC 281)	
	*B. subtilis* (MTCC 441)	*S. aureus* (MTCC 3160)	*E. coli* (MTCC 443)			
**q1**	0.83	0.83	1.65	0.41	1.65	0.18
**q2**	1.55	1.55	1.55	0.78	1.55	3.73
**q3**	1.75	1.75	1.75	0.87	1.75	3.64
**q4**	1.74	1.74	1.74	0.87	1.74	5.02
**q5**	1.80	1.80	1.80	0.90	1.80	2.31
**q6**	1.76	1.76	1.76	0.88	1.76	3.38
**q7**	1.75	1.75	1.75	0.87	1.75	5.59
**q8**	1.68	1.68	1.68	0.84	1.68	2.68
**q9**	1.90	1.90	1.90	1.90	1.90	7.61
**q10**	1.82	1.82	1.82	0.91	1.82	>14.60
**q11**	1.85	1.85	1.85	0.92	1.85	6.79
**q12**	1.90	1.90	1.90	0.95	1.90	7.00
**q13**	1.82	1.82	1.82	0.46	1.82	2.92
**q14**	1.60	1.60	1.60	0.80	1.60	7.16
**q15**	1.60	1.60	1.60	1.60	1.60	>12.79
**q16**	1.54	1.54	0.77	0.77	1.54	11.06
**q17**	1.80	1.80	1.80	0.45	1.80	10.09
**q18**	1.75	1.75	0.87	0.44	1.75	9.79
**q19**	1.63	1.63	0.81	0.41	1.63	11.73
**q20**	1.82	0.36	1.82	0.91	1.82	>14.60
DMSO	0.00	0.00	0.00	0.00	0.00	–
Norfloxacin	0.47	0.47	0.47	–	–	–
Fluconazole	–	–	–	0.50	0.50	–
5-Fluorouracil	–	–	–	–	–	0.35

*Std. drugs* norfloxacin–antibacterial; fluconazole–antifungal; 5-fluorouracil–anticancer

### In vitro anticancer activity

The in vitro anticancer activity of synthesized bis-pyrimidine derivatives was carried out against human colorectal cancer cell line (HCT-116 (ATCC CCL-247) and the results are presented in Table 1. Anticancer screening results revealed that in general bis-pyrimidine Schiff bases exhibited good anticancer potential against human colorectal cancer cell line, especially, compounds **q1** (IC_50_ = 0.18 µmol/mL) displayed anticancer activity more than the reference drug 5-fluorouracil (IC_50_ = 0.35 µmol/L).

### Structure–activity relationship

**Fig. 3:**
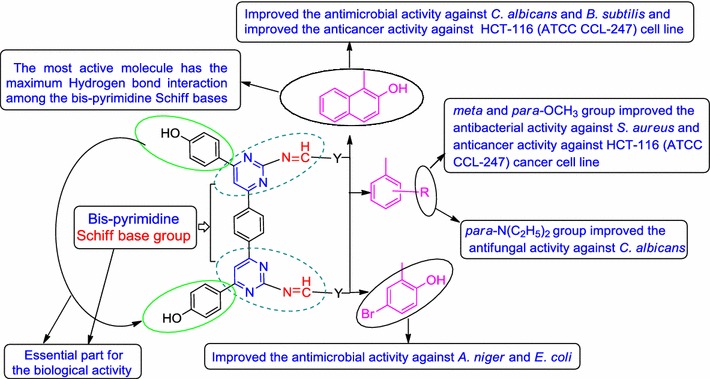
Structural requirements for the antimicrobial and anticancer activities of synthesized bis-pyrimidine Schiff bases

From the antimicrobial and anticancer results, the structure–activity relationship of synthesized bis-pyrimidine Schiff bases (SAR, Fig. 3) can be deduced as follows:

1. Compound **q1** (synthesized using 2-OH naphthaldehyde) was found to be most potent antimicrobial agent against *B. subtilis* and *C. albicans* as well anticancer potential against HCT-116 (ATCC CCL-247) cancer cell line. From the molecular docking studies, compound **q1** being the most active molecule has the maximum hydrogen bond interaction (four) and π–π stacking (three) network among the bis-pyrimidine Schiff bases.
2. Electron withdrawing group [–N(C_2_H_5_)_2_] on benzylidene portion of compound **q19** increased the antifungal potential against *C. albicans.*
3. Presence of electron releasing group (–OCH_3_) on benzylidene portion of compound **q20** enhanced the antibacterial potential against *S. aureus.*
4. Compound **q16** (synthesized using 5-bromo-2-hydroxy benzaldehyde) improved the antimicrobial potential against *A. niger* and *E. coli.*

From the aforementioned results, we may conclude that different structural requirements are required for a compound to be effective against different targets. The aforementioned facts are supported by the earlier research findings [18, 19].

### Docking studies and binding mode analysis

**Fig. 4:**
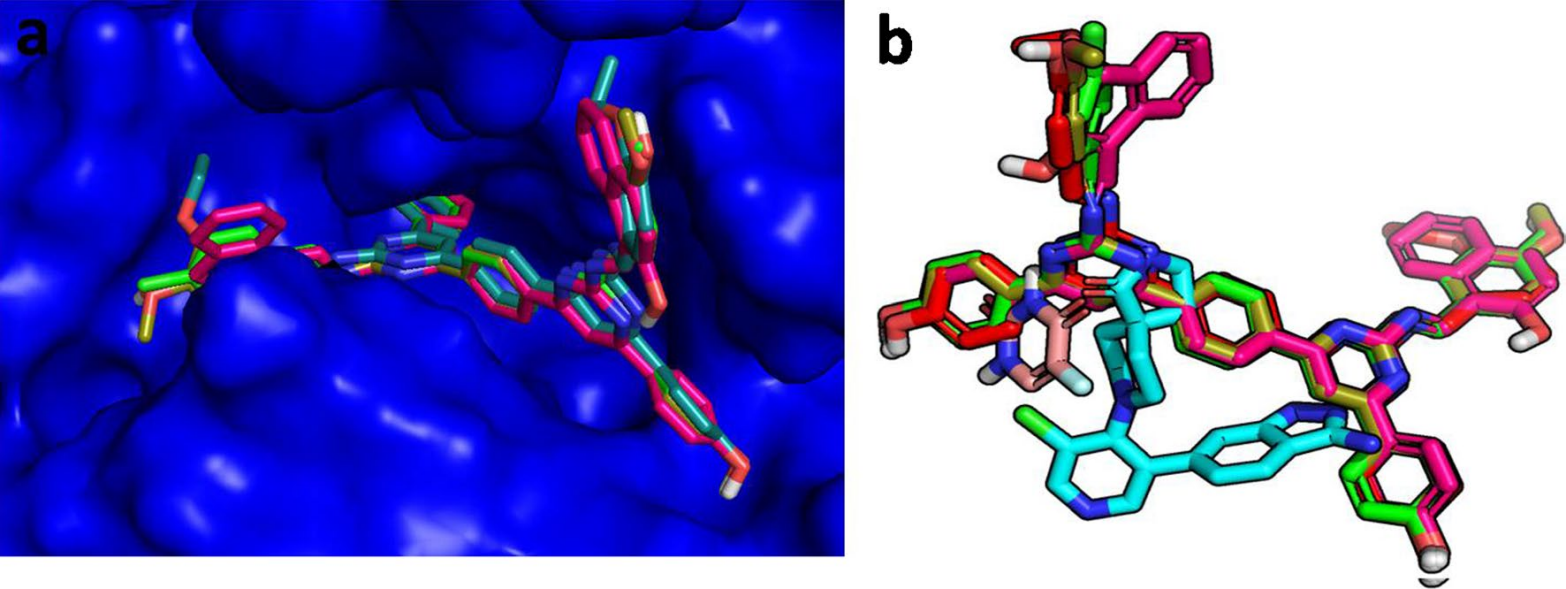
**a** Binding mode of four most active compounds into the CDK8 active site. **b** Overlay of Compound **q1** (magenta color), Compound **q5** (green color), Compound **q8** (red color) and Compound **q13** (split pea color) and PDB Complexed ligand 5XG (color cyan) and 5-Fluorouracil (salmon color) as the reference

Molecular modeling studies were accomplished to investigate the possible binding mode of the synthesized twenty bis-pyrimidine Schiff base derivatives targeting the crystal structure of cyclin-dependent kinase 8 using GOLD docking program. The Schiff bases were docked into the active site of cyclin-dependent kinase CDK8, using co-complex 5XG ligand as the reference with 12 A radius. The results were analyzed based on the ChemPLP scoring function obtained from GOLD. The docked binding mode was analyzed for the interactions between specific compounds and CDK8. Figure 4a shows the binding mode of the active four compounds into the active site of CDK8. While in Fig. 4b shows the binding mode of the co-complexed ligand 5XG and 5-fluorouracil (the standard inhibitor of cancer) is having a different binding mode to that of the four active compounds of bis-pyrimidine Schiff bases. In-depth analysis of the interaction pattern for the most active compounds, **q1**, **q5**, **q8** and **q13** are discussed in the following section.

**Fig. 5:**
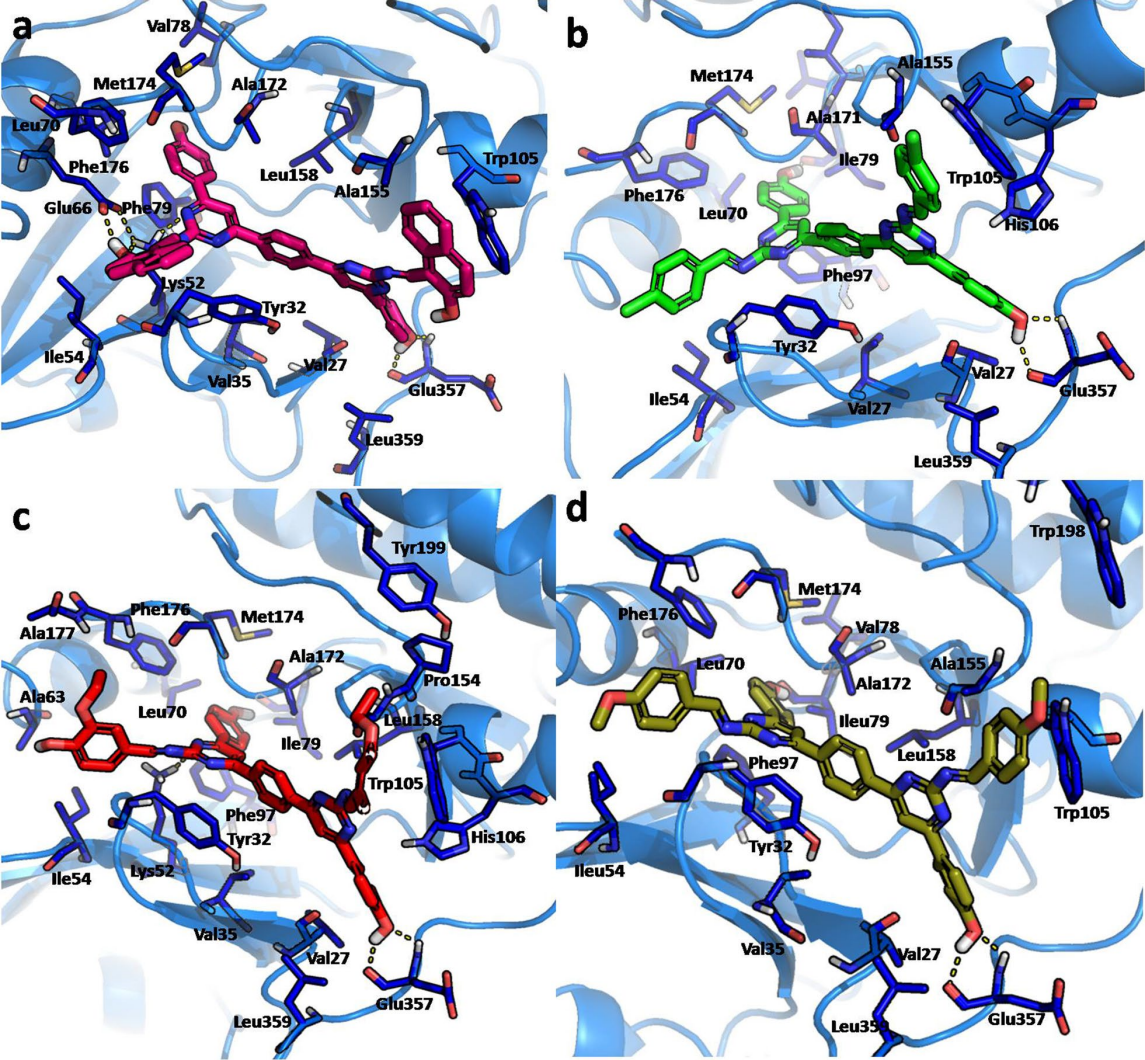
**a** Graphical illustration of predicted binding mode of bis-pyrimidines in the active site of CDK8. **a** Compound **q1** (magenta color), **b** Compound **q5** (green color), **c** Compound **q8** (red color), **d** Compound **q13** (split pea color). Key residues (lines) are only shown and Compounds are represented as sticks. The hydrogen bond interactions are represented by yellow dashed lines

The binding mode of the compound **q1** positioned in the gorge of the CDK8 active site shows that one of the naphthalenol OH of compound **q1** forms hydrogen bond with Glu66 side chain oxygen and with NH of Lys52 side chain, respectively. Additionally, the side chain NH of Lys52 also hydrogen bond with pyrimidinyl nitrogen. While the one of the hydroxyphenyl OH of compound **q1** form a hydrogen bond with side chain oxygen and backbone HN of Glu357. While the Tyr32 phenyl ring forms π–π stacking with phenyl and pyrimidinyl ring of compound **q1** and one of the naphthalene ring also form π–π stacking with indole ring of Trp105. Besides a pool of hydrophobic interaction between compound **q1** and Phe97, Leu70, Ala172, Ile79, Leu158, Met174, Phe176, Ile54, Val35, Val27, Leu359 and Ala155 also stabilize the interaction (Fig. 5a). Compound **1** being the most active compound has the maximum hydrogen bond interaction (four) and π–π stacking (three) network among the bis-pyrimidine Schiff base derivatives. Figure 5b shows the docking orientation of compound **q5**, which is stabilized by the hydrogen bond interaction between one of the hydroxyphenyl OH of compound **q5** forms hydrogen bond with side chain oxygen and backbone HN of Glu357. While the other hydroxyphenyl OH forms hydrogen bond with backbone oxygen of Ile79. Meanwhile, π–π stacking between hydroxyphenyl ring and imidazole ring of His 106, and between chlorophenyl ring and indole ring of Trp105, and π–π stacking between pyrimidinyl ring of compound **q5** and Tyr32 phenyl ring is observed. Additionally hydrophobic contact between compound **q5** and residues such as Val27, Val35, Ala172, Phe97, Leu70, Ile171, Ile79, Val78, Met174, Phe176, Ile54, Leu359 and Ala155 stabilize the complex. While in the case of compound **q5**, is the second most active compound with two hydrogen bond interactions and three π–π stacking network.

In compound **q8**, hydrogen bond interaction between the side chain NH of Lys52 forms hydrogen bond with pyrimidinyl nitrogen and the Glu357 side chain oxygen and backbone HN forms hydrogen bond with hydroxyphenyl OH of compound **q8.** Subsequently, π–π stacking between pyrimidinyl ring of compound **q8** with the Tyr32 phenyl ring and other π–π stacking between hydroxyphenyl rings of compound **q8** with imidazole ring of His106 is observed. Likewise, the presence of aliphatic ethyl group with aromatic rings of compound **q8** forms hydrophobic contacts with Val27, Val35, Ala172, Phe97, Leu70, Ile79, Leu158, Met174, Ile54, Ala63, Phe176, Ala177, Trp105, Pro154, Ala155, Tyr199 and Leu359 is observed (Fig. 5c). While in the case of compound **q8**, is the third most active compound with two hydrogen bond interactions and two π–π stacking interaction.

In the case of compound **q13**, there is a stable hydrogen bond established between the hydroxyphenyl OH with the Glu357 side chain oxygen and backbone HN. While there is the presence of π–π stacking between pyrimidinyl and phenyl ring of compound **q13** with the Tyr32 phenyl ring is noticed. The indole ring of Trp105 forms π–π stacking with the one of the methoxyphenyl ring. Additionally, hydrophobic contact is established between the aromatic groups of compound **q13** with the key hydrophobic residues such as Val27, Val35, Ile54, Phe97, Leu158, Val78, Ala172, Leu70, Ile79, Met174, Phe176, Ala155, Trp198, Leu359 that stabilize the complex (Fig. 5d). whereas in the case of compound **q8**, is the fourth most active compound with two hydrogen bond interactions and two π–π stacking interaction. In the activity profile of the inhibitory assay there is no much difference among compounds, **q5**, **q8** and **q13**. Therefore their binding mode interaction is more or less closer to each other. The hydrogen bonding network and the π–π stacking properties could be the key interactions established by the most active compounds that significantly contribute towards their activity profile. Despite, the fact that the hydrophobic interaction contribution is moderate among the series.

Mutations in adenomatous polyposis coli (APC)/β-catenin resulting in an aberrant activation of Wnt/β-catenin pathway are common in colorectal cancer (CRC), suggesting that targeting the β-catenin pathway with chemopreventive/anticancer agents could be a potential translational approach to control CRC. Recent literature revealed that β-catenin transcriptional activity is positively regulated by the kinase activity of CDK8 and identified it as a CRC oncogene. CDK8, along with cyclin C, Med12, and Med13, forms a “mediator complex” that is involved in the regulation of transcription [20]. The synthesized bispyrimidine Schiff bases may exert their anticancer effect by the inhibition of CDK8 mediated transcription. This was also supported by the observation of Mariaule and Belmont [21] who stated that the pyrimidine is one of the most potential heterocyclic molecules in inhibiting the cyclin dependent kinase as well by the results of molecular docking studies against CDK8 in the current study. Pyrimidines are found to be antagonists of folic acid; hence, a large number of substituted pyrimidines have been synthesized as antifolates and it was eventually proved that these pyrimidines are inhibitors of dihydrofolate reductase (DHFR) [19]. In light of above, the antimicrobial activity of bispyrimidines synthesized in the present study may be attributed to the inhibition of dihydrofolate reductase of the microbe.

### Theoretical ADME prediction of twenty bis-pyrimidine Schiff base derivatives

Theoretical calculations of the ADME (absorption, distribution, metabolism and excretion) properties of synthesized bis-pyrimidine Schiff base derivatives were done using QikPro. Nearly eight physically significant descriptors and pharmacologically relevant properties of the twenty bis-pyrimidine derivatives were predicted and analyzed (Table 2). Aqueous solubility of organic compounds plays a key impact on many ADME associated properties like uptake, distribution, transport, and ultimately bioavailability. The twenty bis-pyrimidine derivatives solubility values were within the range [22]. Finally, the Lipinski’s rule of five and Qikprop rule of three were all within the range for the twenty bis-pyrimidine Schiff bases and thus making these derivatives as suitable drug candidates.

**Table 2:** QikProp ADMET Prediction of twenty bis-pyrimidine derivatives

Comp.	Structure		Comp.	Structure	
**q1**	mol MW: 756.819 Rule of five: 2 Rule of three: 1 Percent human oral absorption: 84.419 QPlogPo/w: 8.778 QPlogBB: −3.759 QPPCaco: 61.3 QPPMDCK: 24.196		**q6**	mol MW: 710.837 Rule of five: 2 Rule of three: 1 Percent human oral absorption: 100.0 QPlogPo/w: 9.148 QPlogBB: −2.863 QPPCaco: 239.969 QPPMDCK: 105.772	
**q2**	mol MW: 804.857 Rule of five: 3 Rule of three: 2 Percent human oral absorption: 84.278 QPlogPo/w: 8.963 QPlogBB: −3.044 QPPCaco: 277.415 QPPMDCK: 123.72		**q7**	mol MW: 714.695 Rule of five: 3 Rule of three: 2 Percent human oral absorption: 40.093 QPlogPo/w: 6.946 QPlogBB: −5.504 QPPCaco: 4.31 QPPMDCK: 1.372	
**q3**	mol MW: 714.695 Rule of five: 3 Rule of three: 2 Percent human oral absorption: 40.146 QPlogPo/w: 6.969 QPlogBB: −5.537 QPPCaco: 4.264 QPPMDCK: 1.357		**q8**	mol MW: 744.805 Rule of five: 3 Rule of three: 1 Percent human oral absorption: 64.212 QPlogPo/w: 7.882 QPlogBB: −4.278 QPPCaco: 47.392 QPPMDCK: 18.321	
**q4**	mol MW: 716.751 Rule of five: 3 Rule of three: 1 Percent human oral absorption: 59 QPlogPo/w: 7.148 QPlogBB: −4.067 QPPCaco: 45.1 QPPMDCK: 17.365		**q9**	mol MW: 656.699 Rule of five: 2 Rule of three: 1 Percent human oral absorption: 66.503 QPlogPo/w: 6.76 QPlogBB: −4.135 QPPCaco: 27.975 QPPMDCK: 10.363	
**q5**	mol MW: 693.59 Rule of five: 2 Rule of three: 1 Percent human oral absorption: 100.0 QPlogPo/w: 9.365 QPlogBB: −2.13 QPPCaco: 304.117 QPPMDCK: 830.136		**q10**	mol MW: 684.753 Rule of five: 2 Rule of three: 1 Percent human oral absorption: 96.326 QPlogPo/w: 8.67 QPlogBB: −2.636 QPPCaco: 307.717 QPPMDCK: 138.39	
**q11**	mol MW: 676.776 Rule of five: 2 Rule of three: 1 Percent human oral absorption: 100.0 QPlogPo/w: 9.671 QPlogBB: −2.961 QPPCaco: 249.332 QPPMDCK: 110.24		**q16**	mol MW: 814.491 Rule of five: 2 Rule of three: 1 Percent human oral absorption: 80.128 QPlogPo/w: 8.109 QPlogBB: −3.358 QPPCaco: 58.418 QPPMDCK: 161.236	
**q12**	mol MW: 656.699 Rule of five: 2 Rule of three: 1 Percent human oral absorption: 71.851 QPlogPo/w: 6.923 QPlogBB: −3.739 QPPCaco: 49.212 QPPMDCK: 19.082		**q17**	mol MW: 693.59 Rule of five: 2 Rule of three: 1 Percent human oral absorption: 100.0 QPlogPo/w: 9.275 QPlogBB: −2.134 QPPCaco: 333.007 QPPMDCK: 613.496	
**q13**	mol MW: 684.753 Rule of five: 2 Rule of three: 1 Percent human oral absorption: 96.551 QPlogPo/w: 8.606 QPlogBB: −2.571 QPPCaco: 332.408 QPPMDCK: 150.43		**q18**	mol MW: 714.695 Rule of five: 3 Rule of three: 2 Percent human oral absorption: 47.45 QPlogPo/w: 7.387 QPlogBB: −5.194 QPPCaco: 7.961 QPPMDCK: 2.664	
**q14**	mol MW: 782.492 Rule of five: 2 Rule of three: 1 Percent human oral absorption: 100.0 QPlogPo/w: 9.567 QPlogBB: −2.067 QPPCaco: 332.297 QPPMDCK: 1056.317		**q19**	mol MW: 766.944 Rule of five: 2 Rule of three: 1 Percent human oral absorption: 100.0 QPlogPo/w: 10.527 QPlogBB: −3.104 QPPCaco: 276.913 QPPMDCK: 123.478	
**q15**	mol MW: 782.492 Rule of five: 2 Rule of three: 1 Percent human oral absorption: 100.0 QPlogPo/w: 9.52 QPlogBB: −2.116 QPPCaco: 304.036 QPPMDCK: 959.515		**q20**	mol MW: 684.753 Rule of five: 2 Rule of three: 1 Percent human oral absorption: 94.52 QPlogPo/w: 8.499 QPlogBB: −2.658 QPPCaco: 277.428 QPPMDCK: 123.726	

## Experimental section

Preparatory materials for the research work were obtained from commercial sources i.e. Loba Chemie, Pvt Ltd. Mumbai, India; Central Drug House (CDH) Pvt. Ltd., New Delhi, India and HiMedia Laboratory Pvt. Ltd., Delhi, India, used without further purification. All reactions were monitored by thin-layer chromatography on 0.25 mm silica gel (Merck) plates, using benzene as mobile phase and spots were observed by exposure to iodine vapours or visualized with UV light. Melting points of synthesized compounds was determined in open capillary tube. An infrared spectrum was recorded (KBr-pellets) in Bruker 12060280, Software: OPUS 7.2.139.1294 spectrometer. ^1^H-NMR and ^13^C-NMR were recorded at 600 and 150 MHz, respectively on Bruker Avance III 600 NMR spectrometer by appropriate deuterated solvents. The results are conveyed in parts per million (*δ*, ppm) downfield from tetramethyl silane (internal standard). ^**1**^H-NMR spectral details of the synthesized derivatives are represented with multiplicity like singlet (s); doublet (d); triplet (t); multiplet (m) and the number hydrogen ion. Elemental analysis of the new synthesized compounds was obtained by Perkin–Elmer 2400 C, H and N analyzer. All the compounds gave C, H and N analysis within ±0.4% of the theoretical results. Mass spectra were taken on Waters Micromass Q-ToF Micro instrument.

### General procedure of the synthesized compounds

#### Step a: synthesis of 3,3′-(1,4-phenylene)bis(1-(4-hydroxyphenyl)prop-2-en-1-one (intermediate-I)

The reaction mixture of 1-(4-hydroxyphenyl)ethanone (0.02 mol) and terephthalaldehyde (0.01 mol) were stirred for 2–3 h in methanol (5–10 mL) followed by drop wise addition of sodium hydroxide solution (10 mL 40%) with constant stirring at room temperature till a dark yellow mass was obtained. Then reaction mixture was allowed to stand overnight at room temperature and then poured into icecold water and acidified with hydrochloric acid and the precipitated 3,3′-(1,4-phenylene)bis(1-(4-hydroxyphenyl)prop-2-en-1-one was filtered, dried and recrystallized from methanol [23].

#### Step b: synthesis of 4,4′-(6,6′-(1,4-phenylene)bis(2-aminopyrimidine-6,4-diyl))diphenol (intermediate-II)

The solution of 3,3′-(1,4-phenylene)bis(1-(4-hydroxyphenyl)prop-2-en-1-one (0.01 mol) (*synthesized in previous step*-*a*) in methanol (80 mL) was added with 0.01 mol of potassium hydroxide and 40 mL of 0.50 M solution of guanidine hydrochloride and refluxed for 5–6 h. The reaction mixture was then cooled and acidified with few drops of hydrochloric acid (20 mL of 0.5 M solution) and the resultant precipitate of 4,4′-(6,6′-(1,4-phenylene)bis(2-amino pyrimidine-6,4-diyl))diphenol was separated out dried and recrystallized from methanol [24].

#### Step c: synthesis of bis-pyrimidine Schiff bases (q1–q20) from intermediate-II (4,4′-(6,6′-(1,4-phenylene)bis(2-aminopyrimidine-6,4-diyl))diphenol)

A mixture of 4,4′-(6,6′-(1,4-phenylene)bis(2-aminopyrimidine-6,4-diyl))diphenol (0.01 mol) (*synthesized in previous step*-*b*) and 0.02 mol of substituted aldehyde was refluxed for 3–4 h in methanol with few drops of glacial acetic acid. The reaction mixture was monitored by thin layer chromatography. After completion of reaction, the reaction mixture was poured into ice cold water and the precipitated title compound was filtered, dried and recrystallized from methanol.

#### Spectral characteristic of the synthesized bis-pyrimidine compounds (q1–q20) and intermediate (I and II)

##### (2E,2′E)-3,3′-(1,4-Phenylene)bis(1-(4-hydroxyphenyl)prop-2-en-1-one) (I)

FT-IR (K–Br, cm^−1^): 3088 (C–H str.), 1427 (C=C str.), 1693 (C=O str.), 1497 (C=C st., alkyl chain), 2864 (C–H sym. str., alkyl chain), 3363 (O–H str., Ar–OH); ^1^H-NMR (DMSO-*d*_6_): 6.91–7.63 (m, 12H, Ar–H), 5.23 (s, 2H, Ar–OH), 7.59 (d, 2H, CH), 8.06 (d, 2H, CH); ^13^C-NMR (δ, DMSO-*d*_6_): 192.92, 164.30, 145.1, 139.69, 134.78, 131.72, 130.21, 129.91, 116.21.

##### 4,4′-(6,6′-(1,4-Phenylene)bis(2-aminopyrimidine-6,4-diyl))diphenol (II)

FT-IR (K–Br, cm^−1^): 3058 (C–H str.), 1537 (C=C str.), 1604 (C=N str. pyrimidine), 3331 (C–N str. –NH_2_), 1388 (C–O st. and OH in pbv); ^1^H-NMR (DMSO-d_6_): 7.37–7.53 (m, 12H, Ar–H), 7.26 (s, 2H, CH of pyrimidine), 3.86 (s, 2H, C–NH_2_); ^13^C-NMR (δ, DMSO-*d*_6_): 193.00, 192.67, 167.12, 139.34, 139.71, 129.71, 129.94, 129.77, 129.30, 128.16, 126.61, 116.16.

##### 1,1′-((1E,1′E)-((6,6′-(1,4-Phenylene)bis(4-(4-hydroxyphenyl)pyrimidinediyl))bis (azanylylidene))bis(methanylylidene))bis(naphthalen-2-ol) (q1)

Light greenish crystals; Yield: 70.45%; mp: 90–92 °C; R_*f*_ value: 0.55; IR (KBr, cm^−1^): 2927 (C–H str.), 1594 (C=C str.), 1698 (N=CH str.), 1313 (C–N str.), 3359 (O–H str.); ^1^H-NMR (δ, DMSO-*d*_6_): 6.96–7.87 (m, 24H, Ar–H), 8.11 (s, 2H, N=CH), 7.70 (s, 2H, (CH)_2_ of pyrimidine); ^13^C-NMR (δ, DMSO-*d*_6_): 163.9, 118.7, 128.7, 124.1, 131.6, 129.2, 118.7, 122.1, 127.5, 112.4; CHN: Calc. C_48_H_32_N_6_O_4_: C, 76.18; H, 4.26; N, 11.10; Found: C, 76.13; H, 4.24; N, 11.12; MS ES + (ToF): *m/z* 758 [M^+^ +1].

##### 4,4′-((E)-6,6′-(1,4-Phenylene)bis(2-((E)-(3,4,5-trimethoxybenzylidene)amino)pyrimidine-6,4-diyl))diphenol (q2)

Dark yellow crystals; Yield: 78.32%; mp: 250–252 °C; R_*f*_ value: 0.15; IR (KBr, cm^−1^): 2830 (C–H str.), 1604 (C=C str.), 1697 (N=CH str.), 1363 (C–N str.), 3352 (O–H str.), 2928 (C–H str., Ar–OCH_3_); ^1^H-NMR (δ, DMSO-*d*_6_): 7.48–7.55 (m, 16H, Ar–H), 8.10 (s, 2H, N=CH), 7.71 (s, 2H, (CH)_2_ of pyrimidine), 3.41{s, 18H, (OCH_3_)_3_}; ^13^C-NMR (δ, DMSO-*d*_6_): 153.2, 129.9, 106.7, 142.8, 131.6, 139.7, 162.6, 167.8,106.7, 150.9, 56.5, 56.2; CHN: Calc. C_46_H_40_N_6_O_8_: C, 68.65; H, 5.01; N, 10.44; Found: C, 68.63; H, 5.04; N, 10.43; MS ES + (ToF): *m/z* 806 [M^+^ +1].

##### 4,4′-((E)-6,6′-(1,4-Phenylene)bis(2-((E)-(4-nitrobenzylidene)amino)pyrimidine-6,4-diyl)) diphenol (q3)

Dark yellow crystals; Yield: 65.34%; mp: 275–277 °C; R_*f*_ value: 0.52; IR (KBr, cm^−1^): 2931 (C–H str.), 1605 (C=C str.), 1700 (N=CH str.), 1301 (C–N str.), 3335 (O–H str.), 1347 (C–NO_2_ sym. str., NO_2_), 1534 (C–NO_2_ asym. str., NO_2_); ^1^H-NMR (δ, DMSO-*d*_6_): 7.04–8.17 (m, 20H, Ar–H), 8.10 (s, 2H, N=CH), 7.70 (s, 2H, (CH)_2_ of pyrimidine); ^13^C-NMR (δ, DMSO-d_6_): 150.5, 129.9, 124.2, 140.0, 130.6, 139.7, 158.5, 116.4, 104.1, 167.8, 160.2, 121.1; CHN: Calc. C_40_H_26_N_8_O_6_: C, 67.22; H, 3.67; N, 15.68; Found: C, 67.24; H, 3.60; N, 15.70; MS ES + (ToF): *m/z* 716 [M^+^ +1].

##### 4,4′-((1E,1′E)-((6,6′-(1,4-Phenylene)bis(4-(4-hydroxyphenyl)pyrimidine-6,2-diyl))bis (azanylylidene))bis(methanylylidene))bis(2-methoxyphenol) (q4)

Dark yellow crystals; Yield: 72.25%; mp: 280–282 °C; R_*f*_ value: 0.54; IR (KBr, cm^−1^): 2933 (C–H str.), 1603 (C=C str.), 1698 (N=CH str.), 1365 (C-N str.), 3337 (O–H str.), 3064 (C–H str., Ar–OCH_3_); ^1^H-NMR (δ, DMSO-*d*_6_): 6.96–7.55 (m, 18H, Ar–H), 8.12 (s, 2H, N=CH), 7.70 (s, 2H, (CH)_2_ of pyrimidine), 3.75 (s, 6H, (OCH_3_)_2_); ^13^C-NMR (δ, DMSO-*d*_6_): 153.1, 148.1, 128.6, 126.0, 115.3, 110.6, 55.5, 158.5, 116.4, 162.5; CHN: Calc. C_42_H_32_N_6_O_6_: C, 70.38; H, 4.45; N, 11.73; Found: C, 70.34; H, 4.40; N, 11.75; MS ES + (ToF): *m/z* 718 [M^+^ +1].

##### 4,4′-((E)-6,6′-(1,4-Phenylene)bis(2-((E)-(4-chlorobenzylidene)amino)pyrimidine-6,4-diyl)) diphenol (q5)

Dark yellow crystals; Yield: 70.25%; mp: 123–125 °C; R_*f*_ value: 0.58; IR (KBr, cm^−1^): 3060 (C–H str.), 1604 (C=C str.), 1700 (N=CH str.), 1384 (C–N str.), 3333 (O–H str.), 776 (C–Cl str. phenyl nucleus); ^1^H-NMR (δ, DMSO-*d*_6_): 6.91–7.61 (m, 20H, Ar–H), 8.10 (s, 2H, N=CH), 7.70 (s, 2H, (CH)_2_ of pyrimidine); ^13^C-NMR (δ, DMSO-*d*_6_): 162.6, 134.7, 131.3, 136.7, 130.1, 129.9, 128.9, 125.6, 115.4; CHN: Calc. C_40_H_26_Cl_2_N_6_O_2_: C, 69.27; H, 3.78; N, 12.12; Found: C, 69.25; H, 3.77; N, 12.09; MS ES + (ToF): *m/z* 695 [M^+^ +1].

##### 4,4′-((E)-6,6′-(1,4-Phenylene)bis(2-((E)-(4-(dimethylamino)benzylidene)amino)pyrimidine-6,4-diyl))diphenol (q6)

Dark yellow crystals; Yield: 72.27%; mp: 250–252 °C; R_*f*_ value: 0.15; IR (KBr, cm^−1^): 2926 (C–H str.), 1595 (C=C str.), 1697 (N=CH str.), 1353 (C–N str.), 3405 (O–H str.), 2830 (N–CH_3_ str.); ^1^H-NMR (δ, DMSO-*d*_6_): 6.78-7.54 (m, 20H, Ar–H), 8.10 (s, 2H, N=CH), 2.89 (s, 12H, (CH_3_)_2_), 7.72 (s, 2H, (CH)_2_ of pyrimidine); ^13^C-NMR (δ, DMSO-*d*_6_): 131.5, 129.9, 124.4, 111.0, 39.9, 158.7, 116.5, 167.1, 144.5, 130.1, 133.1; CHN: Calc. C_44_H_38_N_8_O_2_: C,74.35; H, 5.39; N, 15.76; Found: C, 74.30; H, 5.35; N, 15.72; MS ES + (ToF): *m/z* 712 [M^+^ +1].

##### 4,4′-((E)-6,6′-(1,4-Phenylene)bis(2-((E)-(3-nitrobenzylidene)amino)pyrimidine-6,4-diyl)) diphenol (q7)

Yellow crystals; Yield: 70.82%; mp: 251–253 °C; R_*f*_ value: 0.54; IR (KBr, cm^−1^): 2927 (C–H str.), 1629 (C=C str.), 1698 (N=CH str.), 1352 (C–N str.), 3386 (O–H str.), 1602 (NO_2_ str.), 814 (C–N str., –NO_2_); ^1^H-NMR (δ, DMSO-*d*_6_): 7.50-8.69 (m, 20H, Ar–H), 8.10 (s, 2H, N=CH), 7.70 (s, 2H, (CH)_2_ of pyrimidine); ^13^C-NMR (δ, DMSO-*d*_6_): 139.7, 137.1, 134.8, 130.9, 129.9, 128.5, 126.5, 124.0; CHN: Calc. C_40_H_26_N_8_O_6_: C, 67.22; H, 3.67; N, 15.68; Found: C, 67.20; H, 3.70; N, 15.63; MS ES + (ToF): *m/z* 716 [M^+^ +1].

##### 4,4′-((1E,1′E)-((6,6′-(1,4-Phenylene)bis(4-(4-hydroxyphenyl)pyrimidine-6,2-diyl))bis(azanyl ylidene))bis(methanylylidene))bis(2-ethoxyphenol) (q8)

Yellow crystals; Yield: 64.33%; mp: 260–262 °C; R_*f*_ value: 0.25; IR (KBr, cm^−1^): 2928 (C–H str.), 1597 (C=C str.), 1629 (N=CH str.), 1353 (C–N str.), 3408 (O–H str.), 2830 (C–H str.,–OC_2_H_5_); ^1^H-NMR (δ, DMSO-*d*_6_): 6.90–7.53 (m, 18H, Ar–H), 8.10 (s, 2H, N=CH), 7.72 (s, 2H, (CH)_2_ of pyrimidine), 4.08 {q, 4H, (CH_2_)_2_, 1.34 (t, 6H, (CH_3_)_2_}; ^13^C-NMR (δ, DMSO-*d*_6_): 147.4, 131.3, 129.9, 128.0, 126.0, 115.5, 111.7, 63.7, 14.5; CHN: Calc. C_44_H_36_N_6_O_6_: C, 70.96; H, 4.87; N, 11.28; Found: C, 70.91; H, 4.83; N, 11.23; MS ES + (ToF): *m/z* 746 [M^+^ +1].

##### 4,4′-((E)-6,6′-(1,4-Phenylene)bis(2-((E)-(4-hydroxybenzylidene)amino)pyrimidine-6,4-diyl)) diphenol (q9)

Yellow crystals; Yield: 73.45%; mp: 200–202 °C; R_*f*_ value: 0.16; IR (KBr, cm^−1^): 2927 (C–H str.), 1509 (C=C str.), 1697 (N=CH str.), 1354 (C–N str.), 3387 (O–H str.); ^1^H-NMR (δ, DMSO-*d*_6_): 6.91–7.53 (m, 20H, Ar–H), 8.10 (s, 2H, N=CH), 7.72 (s, 2H, (CH)_2_ of pyrimidine); ^13^C-NMR (δ, DMSO-*d*_6_): 139.7, 132.0, 131.3, 129.9, 115.0, 158.1, 116.4, 162.4, 104.1, 133.1, 167.5, 160.1; CHN: Calc. C_40_H_28_N_6_O_4_: C,73.16; H, 4.30; N, 12.80; Found: C, 73.13; H, 4.34; N, 12.84; MS ES + (ToF): *m/z* 658 [M^+^ +1].

##### 4,4′-((E)-6,6′-(1,4-Phenylene)bis(2-((E)-(2-methoxybenzylidene)amino)pyrimidine-6,4-diyl)) diphenol (q10)

Yellow crystals; Yield: 77.34%; mp: 244–246 °C; R_*f*_ value: 0.33; IR (KBr, cm^−1^): 2830 (C–H str.), 1540 (C=C str.), 1604 (N=CH str.), 1353 (C–N str.), 3357 (O–H str.), 2931(C–H str., Ar–OCH_3_); ^1^H-NMR (δ, DMSO-*d*_6_): 7.00–7.55 (m, 20H, Ar–H), 8.12 (s, 2H, N=CH), 7.70 (s, 2H, (CH)_2_ of pyrimidine), 3.58 {s, 6H, (OCH_3_)_2_}; ^13^C-NMR (δ, DMSO-*d*_6_): 129.9, 127.6, 120.5, 55.8, 158.5, 116.4, 125.7, 167.8, 130.2, 132.1, 55.9; CHN: Calc. C_42_H_32_N_6_O_4_: C, 73.67; H, 4.71; N, 12.27; Found: C, 73.63; H, 4.74; N, 12.22; MS ES + (ToF): *m/z* 686 [M^+^ +1].

##### 4,4′-(6,6′-(1,4-Phenylene)bis(2-((E)-((E)-3-phenylallylidene)amino)pyrimidine-6,4-diyl)) diphenol (q11)

Yellow crystals; Yield: 62.33%; mp: 250–252 °C; R_*f*_ value: 0.45; IR (KBr, cm^−1^): 3059 (C–H str.), 1538 (C=C str.), 1699 (N=CH str.), 1386 (C–N str.), 3330 (O–H str.), 2932 (C–H str., aliphatic), 1606 (C=C str., alkyl chain); ^1^H-NMR (δ, DMSO-*d*_6_): 6.85–7.53 (m, 22H, Ar–H), 7.75 (s, 2H, N=CH), 7.7 (s, 2H, (CH)_2_ of pyrimidine); ^13^C-NMR (δ, DMSO-*d*_6_): 131.1, 129.9, 128.6, 128.4, 158.5, 116.4, 125.7, 162.5, 163.6, 138.2, 126.4, 135.4; CHN: Calc. C_44_H_32_N_6_O_2_: C, 78.09; H, 4.77; N, 12.42; Found: C, 78.05; H, 4.72; N, 12.39; MS ES + (ToF): *m/z* 678 [M^+^ +1].

##### 4,4′-((E)-6,6′-(1,4-Phenylene)bis(2-((E)-(2-hydroxybenzylidene)amino)pyrimidine-6,4-diyl)) diphenol (q12)

Yellow crystals; Yield: 81.84%; mp: 195–197 °C; R_*f*_ value: 0.80; IR (KBr, cm^−1^): 2930 (C–H str.), 1540 (C=C str.), 1699 (N=CH str.), 1354 (C–N str.), 3372 (O–H str.); ^1^H-NMR (δ, DMSO-*d*_6_): 6.96–7.54 (m, 20H, Ar–H), 8.10 (s, 2H, N=CH), 7.72 (s, 2H, (CH)_2_ of pyrimidine); ^13^C-NMR (δ, DMSO-*d*_6_): 129.9, 158.5, 116.4, 128.9, 125.7,162.5, 167.8, 118.5, 130.6, 121.5, 132.1, 161.4; CHN: Calc. C_40_H_28_N_6_O_4_: C, 73.16; H, 4.30; N, 12.80; Found: C, 73.13; H, 4.34; N, 12.78; MS ES + (ToF): *m/z* 658 [M^+^ +1].

##### 4,4′-((E)-6,6′-(1,4-Phenylene)bis(2-((E)-(4-methoxybenzylidene)amino)pyrimidine-6,4-diyl)) diphenol (q13)

Yellow crystals; Yield: 89.14%; mp: 255–257 °C; R_*f*_ value: 0.32; IR (KBr, cm^−1^): 2830 (C–H str.), 1540 (C=C str.), 1699 (N=CH str.), 1354 (C–N str.), 3359 (O–H str.), 2931 (C–H str., Ar–OCH_3_); ^1^H-NMR (δ, DMSO-*d*_6_): 7.12–7.59 (m, 20H, Ar–H), 8.10 (s, 2H, N=CH), 7.68 (s, 2H, (CH)_2_ of pyrimidine), 3.74 (s, 6H, OCH_3_); ^13^C-NMR (δ, DMSO-*d*_6_): 131.7, 129.9, 114.4, 125.8, 162.6, 104.3, 167.8, 160.1, 128.0, 133.1, 158.1, 116.4, 126.1, 163.0, 55.6; CHN: Calc. C_42_H_32_N_6_O_4_: C, 73.67; H, 4.71; N, 12.27; Found: C, 73.62; H, 4.75; N, 12.31; MS ES + (ToF): *m/z* 686 [M^+^ +1].

##### 4,4′-((E)-6,6′-(1,4-Phenylene)bis(2-((E)-(3-bromobenzylidene)amino)pyrimidine-6,4-diyl)) diphenol (q14)

Yellow crystals; Yield: 66.90%; mp: 205–207 °C; R_*f*_ value: 0.27; IR (KBr, cm^−1^): 3057 (C–H str.), 1538 (C=C str.), 1699 (N=CH str.), 1387 (C–N str.), 3329 (O–H str.), 549 (C–Br str.); ^1^H-NMR (δ, DMSO-*d*_6_): 7.36–782 (m, 20H, Ar–H), 8.12 (s, 2H, N=CH), 7.72 (s, 2H, (CH)_2_ of pyrimidine); ^13^C-NMR (δ, DMSO-*d*_6_): 139.7, 137.0, 131.9, 128.1, 122.3, 158.5, 116.3, 167.6, 134.0, 162.3, 104.1; CHN: Calc. C_40_H_26_Br_2_N_6_O_2_: C, 61.40; H, 3.35; N, 10.74; Found: C, 61.45; H, 3.30; N, 10.77; MS ES + (ToF): *m/z* 783 [M^+^ +1].

##### 4,4′-((E)-6,6′-(1,4-Phenylene)bis(2-((E)-(4-bromobenzylidene)amino)pyrimidine-6,4-diyl)) diphenol (q15)

Yellow crystals; Yield: 72.00%; mp: 217–219 °C; R_*f*_ value: 0.58; IR (KBr, cm^−1^): 3059 (C–H str.), 1538 (C=C str.), 1699 (N=CH str.), 1386 (C–N str.), 3338 (O–H str.), 1168 (C–O–C str., Ar–OCH_3_), 2830 (C–H str., –OCH_3_), 551 (C–Br str.); ^1^H-NMR (δ, DMSO-*d*_6_): 7.46–7.53 (m, 20H, Ar–H), 8.12 (s, 2H, N=CH), 7.70 (s, 2H, (CH)_2_ of pyrimidine); ^13^C-NMR (δ, DMSO-*d*_6_): 139.7, 132.2, 131.1, 128.6, 126.5, 158.5, 116.4, 131.8, 125.3, 167.8, 160.2; CHN: Calc. C_40_H_26_Br_2_N_6_O_2_: C, 61.40; H, 3.35; N, 10.74; Found: C, 61.45; H, 3.30; N, 10.77; MS ES + (ToF): *m/z* 783 [M^+^ +1].

##### 2,2′-((1E,1′E)-((6,6′-(1,4-Phenylene)bis(4-(4-hydroxyphenyl)pyrimidine-6,2-diyl))bis(azanyl ylidene))bis(methanylylidene))bis(4-bromophenol) (q16)

Yellow crystals; Yield: 83.67%; mp: 296–298 °C; R_*f*_ value: 0.25; IR (KBr, cm^−1^): 542 (C–Br str., C_6_H_5_Br), 2928 (C–H str.), 1604 (C=C str.), 1699 (N=CH str.), 1363 (C–N str.), 3357 (O–H str.); ^1^H-NMR (δ, DMSO-*d*_6_): 7.39–7.61 (m, 18H, Ar–H), 8.11 (s, 2H, N=CH), 7.71 (s, 2H, (CH)_2_ of pyrimidine); ^13^C-NMR (δ, DMSO-*d*_6_): 158.4, 163.6, 104.1, 167.8, 160.1, 128.0, 130.0, 120.4, 133.1, 115.8, 116.4, 128.9, 125.7, 135.4, 118.2, 160.1; CHN: Calc. C_40_H_26_Br_2_N_6_O_4_: C, 58.99; H, 3.22; N, 10.32; Found: C, 58.95; H, 3.20; N, 10.35; MS ES + (ToF): *m/z* 815 [M^+^ +1].

##### 4,4′-((E)-6,6′-(1,4-Phenylene)bis(2-((E)-(2-chlorobenzylidene)amino)pyrimidine-6,4-diyl)) diphenol) (q17)

Yellow crystals; Yield: 79.12%; mp: 265–267 °C; R_*f*_ value: 0.18; IR (KBr, cm^−1^): 2925 (C–H str.), 1539 (C=C str.), 1699 (N=CH str.), 1363 (C–N str.), 3355 (O–H str.), 775(C–Cl str.); ^1^H-NMR (δ, DMSO-*d*_6_): 7.48–7.61 (m, 20H, Ar–H), 8.12 (s, 2H, N=CH), 7.70 (s, 2H, (CH)_2_ of pyrimidine); ^13^C-NMR (δ, DMSO-*d*_6_): 139.7, 135.7, 130.7, 129.7, 127.8; CHN: Calc. C_40_H_26_Cl_2_N_6_O_2_: C, 69.27; H, 3.78; N, 12.12; Found: C, 69.23; H, 3.74; N, 12.10; MS ES + (ToF): *m/z* 695 [M^+^ +1].

##### 4,4′-((E)-6,6′-(1,4-Phenylene)bis(2-((E)-(2-nitrobenzylidene)amino)pyrimidine-6,4-diyl)) diphenol (q18)

Yellow crystals; Yield: 69.23%; mp: 198–200 °C; R_*f*_ value: 0.16; IR (KBr, cm^−1^): 2926 (C–H str.), 1539 (C=C str.), 1699 (N=CH str.), 1354 (C–N str.), 3375 (O–H str.), 1604 (NO_2_ str.), 814 (C–N str., –NO_2_); ^1^H-NMR (δ, DMSO-*d*_6_): 7.43–8.16 (m, 20H, Ar–H), 8.12 (s, 2H, N=CH), 7.75 (s, 2H, (CH)_2_ of pyrimidine); ^13^C-NMR (δ, DMSO-*d*_6_): 167.4, 139.7, 134.1, 130.5, 128.1, 126.5, 124.2; CHN: Calc. C_40_H_26_N_8_O_6_: C, 67.22; H, 3.67; N, 15.68; Found: C, 67.18; H, 3.62; N, 15.63; MS ES + (ToF): *m/z* 716 [M^+^ +1].

##### 4,4′-((E)-6,6′-(1,4-Phenylene)bis(2-((E)-(4-(diethylamino)benzylidene)amino)pyrimidine-6,4-diyl))diphenol (q19)

Light yellow crystals; Yield: 65.33%; mp: 249–251 °C; R_*f*_ value: 0.23; IR (KBr, cm^−1^): 2970 (C–H str.), 1590 (C=C str.), 1699 (N=CH str.), 1356 (C–N str.), 2830 (C–H str., alkyl chain), 3350 (O–H str.); ^1^H-NMR (δ, DMSO-*d*_6_): 6.75–7.55 (m, 20H, Ar–H), 8.1 (s, 2H, N=CH), 7.77 (s, 2H, (CH)_2_ of pyrimidine), 3.41 {q, 4H, (CH_2_)_2_, 1.13 (t, 6H, (CH_3_)_2_}; ^13^C-NMR (δ, DMSO-*d*_6_): 151.9, 139.7, 129.9, 123.9, 110.5, 116.4, 126.9, 160.1, 128.0, 133.1, 130.1, 125.7, 162.6, 104.1, 167.8, 114.4, 44.5, 12.27; CHN: Calc. C_48_H_46_N_8_O_2_: C, 75.17; H, 6.05; N, 14.61; Found; C,75.14; H, 6.09; N, 14.64; MS ES + (ToF): *m/z* 768 [M^+^ +1].

##### 4,4′-((E)-6,6′-(1,4-Phenylene)bis(2-((E)-(2-methoxybenzylidene)amino)pyrimidine-6,4-diyl)) diphenol (q20)

Light yellow crystals; Yield: 89.45%; mp: 274–276 °C; R_*f*_ value: 0.16; IR (KBr, cm^−1^): 2931 (C–H str.), 1605 (C=C str.), 1699 (N=CH str.), 1365 (C–N str.), 3350 (O–H str.), 3058 (C–H str.,–OCH_3_); ^1^H-NMR (δ, DMSO-*d*_6_): 6.91–7.54 (m, 20H, Ar–H), 8.1 (s, 2H, N=CH), 7.62 (s, 2H, (CH)_2_ of pyrimidine), 3.75 (s, 2H, OCH_3_); ^13^C-NMR (δ, DMSO-*d*_6_): 159.1, 130.3, 129.9, 129.1, 128.1, 122.4, 120.9, 118.7, 113.8, 116.3, 126.9, 125.7, 163.6, 104.1, 134.8, 121.5, 128.8, 160.8, 55.3; CHN: Calc. C_42_H_32_N_6_O_4_: C, 73.67; H, 4.71; N, 12.27; Found: C, 73.62; H, 4.75; N, 12.22; MS ES + (ToF): *m/z* 686 [M^+^ +1].

### In vitro antimicrobial assay

The in vitro antimicrobial study of the synthesized bis-pyrimidines was evaluated against Gram +ve bacterial species: *S. aureus* (MTCC 3160), *B. subtilis* (MTCC 441), Gram −ve species: *E. coli* (MTCC 443) and fungus species: *A. niger* (MTCC 281) and *C. albicans* (MTCC 227) by tube dilution technique [25]. Dilutions of test and reference drug in double strength nutrient broth media I.P. was used for antibacterial study and Sabouraud dextrose broth media I.P. was used for the antifungal study. The stock solution was prepared for the test compounds (**q1**–**q20**) and reference drugs (norfloxacin and fluconazole) in dimethyl sulfoxide (DMSO) to get a concentration of 100 µg/mL and this stock solution was used for further tube dilution with six concentration of 50, 25, 12.5, 6.25, 3.125 and 1.562 µg/mL for the antimicrobial study [26]. The MIC values of synthesized bis-pyrimidine Schiff base derivatives were recorded at different incubation period: 37 ± 1 °C (bacterial species) for 24 h, 37 ± 1 °C (*C. albicans*) for 48 h and 25 ± 1 °C (*A. niger*) for 7 days and the antimicrobial results have been recorded in terms of minimum inhibitory concentration values in µmol/mL.

### In vitro cytotoxicity assay

The anticancer screening of synthesized compounds was determined against human colorectal carcinoma [HCT-116 (ATCC (American Type Culture Collection) CCL-247)] cancer cell line using sulforhodamine B (SRB) assay. The optimal cell count (2500 cells/180 μL/well) of HCT-116 was seeded onto the 96 flat-bottom well plates and incubated overnight to allow attachment. 20 μL of pure compounds at tenfold the final concentrations were added in quadruplicates. Both drug-free control and treated cells were then incubated for 72 h. The drug-induced cytotoxicity was assessed using the SRB assay as previously described by Skehan et al. [27], but with minor modifications. Briefly, upon removal of media, cells in each well were fixed with 200 μL of 10% cold TCA [Sigma-Aldrich, St Louis, Missouri, USA] (w/v; in deionised water). After incubation at 4 0 °C for 30 min, the individual wells were rinsed with water for five times. Cells in each well were allowed to stain in 100 µL of 0.4% SRB [Sigma-Aldrich, St Louis, Missouri, USA] (w/v; in 1% acetic acid) for 15 min. Unincorporated dye was rinsed off with 1% acetic acid [Fisher Scientific, Loughborough, Leicestershire, UK] (v/v; in deionised water) and plates were left to air-dry at room temperature overnight. The air-dried plates were placed on a plate shaker and bound SRB was solubilised in 100 µL of 10 mM Tris base solution [Sigma-Aldrich, St Louis, Missouri, USA]. Absorbance was measured by a computer-interfaced 96-well plate spectrophotometer at 570 nm. A dose–response curve (percentage of cell viability vs log concentration) was plotted from which the IC50 value of each molecule against each cell type was graphically determined.

### Molecular docking protocol

In order to reveal the binding modes of synthesized twenty bis-pyrimidine Schiff base derivatives, docking simulation was performed targeting the crystal structure of cyclin-dependent kinase 8 (CDK8). Prior to docking, the crystal structure [PDB ID: 5FGK] was retrieved from the protein data bank (PDB) [28]. The CDK8 structure was prepared using protein preparation wizard and optimized by removing the water molecules, hetero atoms and co-factors. Hydrogen, missing atoms, bonds and charges were computed through Maestro [Schrodinger Release 2015-1: Maestro, version 10.1, Schrodinger, LLC, New York, 2015]. The synthesized twenty.

Bis-pyrimidine Schiff base derivatives were used for docking. Meanwhile, the bis-pyrimidine Schiff base derivatives were prepared and optimized using built and LigPrep module implemented in Schrodinger Maestro. Ligands preparation includes generating various tautomers, assigning bond orders, ring conformations and stereo chemistries. All the conformations generated were minimized using OPLS2005 force field prior to docking study.

Molecular docking studies were performed using GOLD (Genetic Optimization for Ligand Docking) program version 5.1. GOLD is an automated docking program that employs the genetic algorithm to search the ligand conformational flexibility with a partial flexibility of protein’s active site [29]. GOLD uses genetic algorithm method for protein–ligand docking and it is well-known for its performance and accuracy specifically for the protein targets with buried active site. The GOLD software has four scoring functions namely ChemPLP, GoldScore, ChemScore, and ASP (the Astex Statistical Potential) which take into account the terms of hydrogen bonding, van der Waal and intramolecular energies. In the present GOLD docking study targeting cyclin-dependent kinase CDK8, the ChemPLP scoring function was used as it outperformed the other scoring function. All the bis-pyrimidine derivatives were docked to cyclin-dependent kinase CDK8 active site, using co-complex 5XG ligand as the reference with 12 A radius. Further, the population size was set to (100); selection-pressure (1.1); number of operations (10,000); number of islands (1); niche size (2); operator weights for migrate (0), mutate (100), and cross-over (100) and with 100 GA run. Results divergent by less than 1.50 A in ligand-all atom RMSDs were clustered together. Best cluster poses and top ranked scores were saved and visually analyzed by Pymol [PyMOL Molecular Graphics System, Schrödinger L, NY, USA, 2010.]. Additional, Qikprop prediction of ADME properties were done for all the synthesized twenty bis-pyrimidine derivatives [Rapid ADME, QikProp, Schrödinger LLC, New York, 2012].

## Conclusion

Summarizingly, bis-pyrimidine Schiff bases were synthesized and characterized by physicochemical and spectral means and the spectral data was found in agreement with the assigned molecular structures. The in vitro antimicrobial screening of synthesized compounds indicated that compounds **q1**, **q16**, **q19** and **q20** exhibited appreciable antimicrobial potential. The anticancer screening results demonstrated that compound **q1 (**IC_50_ = 0.18 µmol/mL**)** is the most active one against colorectal (HCT 116) cancer cell line. Molecular docking studies indicated the compound **q1** being the most active molecule has the maximum hydrogen bond interaction (four) and π–π stacking (three) network among the bis-pyrimidine Schiff bases. The synthesized compounds may exhibit their anticancer and antimicrobial activity by the inhibition of enzymes CDK-8 and DHFR respectively. The current study has widened the scope of developing the most active bis-pyrimidine molecules as promising antimicrobial and anticancer agents.
